# Conservative kidney management in a young patient with severe intellectual disability: clinical implications for kidney therapy decision-making - a case report

**DOI:** 10.1186/s12882-026-05106-6

**Published:** 2026-06-25

**Authors:** Hajime Hirano, Tomohisa Matsunaga, Takahiro Inoue, Yu Munakata, Tetsuro Machimura, Tatsuhiko Mori, Haruhito Azuma, Norio Hanafusa

**Affiliations:** 1 Department of Blood Purification, Tokyo Women’s Medical University, 8-1 Kawada-cho, Shinjuku-ku, Tokyo, 162-8666 Japan; 2 Blood Purification Center, Osaka Medical and Pharmaceutical University Hospital, Osaka, Japan; 3 Department of Nephrology, Osaka Medical and Pharmaceutical University, Osaka, Japan

**Keywords:** Conservative kidney management, Advanced chronic kidney disease, Intellectual disability, Decision-making capacity, Shared decision-making, Ethics

## Abstract

**Background:**

Conservative kidney management (CKM) is increasingly recognized as a patient-centered treatment pathway for individuals with advanced chronic kidney disease (CKD). However, most CKM frameworks assume that patients can participate in shared decision-making, and little guidance exists for younger patients with a lifelong severe intellectual disability who lack decision-making capacity.

**Case presentation:**

We report the case of a young adult with advanced CKD secondary to congenital anomalies of the kidney and urinary tract and severe intellectual disability who lacked decisional capacity. As kidney failure progressed, all modalities of kidney replacement therapy, including hemodialysis, peritoneal dialysis, and kidney transplantation, were considered. However, concerns regarding treatment feasibility, caregiver burden, and anticipated impact on quality of life led to the selection of CKM after repeated multidisciplinary discussions involving clinicians and family members. Home-based medical care and visiting nursing services were introduced to support symptom management and family caregivers. The patient remained on CKM without dialysis initiation and died peacefully at home 35 months later, surrounded by family.

**Conclusions:**

This case highlights the ethical and clinical challenges of kidney therapy decision-making in patients with severe intellectual disability who cannot participate in shared decision-making. CKM may represent an ethically and clinically appropriate care pathway when dialysis is technically feasible but unlikely to improve quality of life, minimize treatment burden, or align with the patient’s best interests as determined through multidisciplinary deliberation.

## Background

Conservative kidney management (CKM) is increasingly recognized as a legitimate treatment pathway for patients with advanced chronic kidney disease (CKD), particularly among older adults and those with substantial comorbidity. Growing evidence suggests that, in selected populations, CKM may provide acceptable survival with reduced treatment burden and hospitalization compared with dialysis-centered care [1–3]. To our knowledge, this is among the first reported cases in which CKM was selected for a young adult with a lifelong severe intellectual disability who lacked decision-making capacity, a population systematically excluded from existing CKM frameworks.

Despite this progress, existing CKM frameworks largely assume that patients can participate meaningfully in shared decision-making (SDM). In clinical practice, however, nephrologists increasingly encounter patients who lack decisional capacity due to cognitive impairment, neurodevelopmental disorders, or severe psychiatric illness. In such cases, dialysis may be technically feasible yet poorly aligned with patient-centered goals, raising complex ethical and practical challenges.

Recent literature has emphasized the need to reconceptualize SDM in kidney failure as an iterative process that can incorporate surrogate decision-making, uncertainty, and evolving goals of care [4, 5]. Ethical analyses and clinical practice guidelines further recognize that dialysis initiation is not obligatory when anticipated burdens outweigh potential benefits, and that CKM should be presented as a valid alternative within patient-centered care.

Nevertheless, there remains limited guidance on how to operationalize CKM for younger patients with a lifelong severe intellectual disability who lack decision-making capacity, in whom the long-term implications of dialysis-related burden may be particularly profound.

Here, we report a case of a young adult with advanced CKD and severe intellectual disability in whom CKM was selected after extensive multidisciplinary and family-centered deliberation. Beyond the rarity of the clinical scenario, this case highlights a broader and increasingly relevant challenge in nephrology: situations in which renal replacement therapy is technically feasible but unlikely to improve quality of life or align with the patient’s best interests. By describing the ethical reasoning and multidisciplinary process that led to CKM selection, this report aims to provide practical insights for clinicians facing similar decision-making dilemmas.

## Case presentation

The patient was a 38-year-old man with advanced chronic kidney disease (CKD) secondary to congenital anomalies of the kidney and urinary tract, in the context of Down syndrome (Trisomy 21). The patient had been followed by pediatric nephrology services during childhood and adolescence and was transitioned to adult nephrology care at age 19 as his condition stabilized. Kidney function had remained relatively stable for many years thereafter, with gradual progression of dysfunction becoming apparent in adulthood.

The patient had severe intellectual disability. A formal psychiatric evaluation confirmed a mental age of approximately 1 year and an intelligence quotient below 20, consistent with profound intellectual impairment. He was unable to communicate verbally or understand complex information. However, he was able to express basic preferences through non-verbal behaviors, such as facial expressions and gestures, particularly in response to pleasurable or distressing stimuli. He was unable to comprehend medical information or participate in decision-making regarding treatment.

He lived with his parents and attended a daytime workshop on weekdays, which he appeared to enjoy and which constituted a central component of his daily life. His functional status was stable, and he was able to perform basic activities such as eating and bathing with supervision. However, he demonstrated strong behavioral rigidity and limited adaptability to unfamiliar environments. For example, he consistently refused medication unless administered by his mother and would remove medical devices such as dressings or tapes immediately after application.

As kidney function declined (estimated glomerular filtration rate approximately 15 mL/min/1.73 m²), discussions regarding kidney replacement therapy (KRT) were initiated. Given the patient’s lifelong lack of decisional capacity, treatment decisions were made through a surrogate decision-making process involving his parents, who were his primary caregivers, as well as multidisciplinary healthcare professionals including nephrologists, nurses, mental health specialists, and medical social workers. In accordance with Japanese clinical practice, surrogate decision-making was conducted by the patient’s parents, who served as his primary caregivers, with consensus from extended family members. Formal legal guardianship had not been established; however, the decision-making process followed accepted ethical standards and institutional practice for patients lacking decisional capacity.

All KRT modalities were carefully evaluated. Hemodialysis was considered technically feasible; however, the patient was unable to remain still during procedures, raising concerns that repeated sedation would be required to safely perform vascular access cannulation and dialysis sessions. This raised substantial concerns regarding both safety and long-term feasibility. In addition, adherence to vascular access care and thrice-weekly hospital visits were considered impractical given his behavioral characteristics and dependence on caregivers.

Peritoneal dialysis was also assessed; however, the patient demonstrated a high likelihood of removing catheters or interfering with dialysis equipment. He was unable to understand or cooperate with dialysis procedures, and caregiver-dependent management would be required continuously. Furthermore, acceptance of assistance from individuals other than his mother was limited, raising concerns regarding sustainability of care.

Kidney transplantation was discussed as a potentially definitive therapy. However, several critical barriers were identified. The patient was unable to tolerate preoperative and perioperative management, including invasive monitoring and catheter placement. More importantly, adherence to lifelong immunosuppressive therapy was considered unachievable. A trial of medication administration simulating post-transplant regimens confirmed significant difficulty even under controlled conditions. These factors raised a substantial risk of graft loss and subsequent need for dialysis.

Throughout the decision-making process, repeated multidisciplinary meetings were conducted. The patient’s parents initially expressed a preference for life-prolonging treatment, including transplantation. However, through iterative discussions that carefully weighed the expected benefits and burdens of each modality, the family came to prioritize the patient’s quality of life, particularly the preservation of his daily routine and avoidance of distressing medical interventions.

To ensure a robust surrogate decision-making process, extended family members, including siblings, were also involved in discussions, and consensus was achieved. The decision-making process was further supported by psychiatric assessment of decisional capacity and consultation with relevant ethical and professional frameworks, including the Guidelines for Discussions on Medical Care for Children with Serious Illness published by the Japan Pediatric Society (revised 2024) and the Japanese Ministry of Health, Labour and Welfare Guidelines on the Decision-Making Process for Medical Care at the End of Life.

Ultimately, conservative kidney management (CKM) was selected as the most appropriate treatment approach, based on a best-interest standard that considered the patient’s inability to benefit meaningfully from KRT and the high likelihood of treatment-related burden.

Following this decision, a care plan was established to support ongoing quality of life. This included regular outpatient monitoring, symptom-directed management, early integration of home-based medical care, and visiting nursing services. Particular attention was given to maintaining continuity of the patient’s daily activities and minimizing environmental disruption.

During the course of conservative kidney management, the patient developed symptoms consistent with advanced kidney failure, including progressive fatigue, decreased appetite, and intermittent lower extremity edema. Episodes suggestive of uremic symptoms, such as reduced activity levels and increased somnolence, were also observed, although formal symptom reporting was limited due to his inability to communicate verbally. Anemia was managed conservatively, and no invasive interventions such as transfusion were pursued in accordance with the agreed goals of care. Symptom-oriented management was prioritized, including careful fluid balance, dietary adjustments, and home-based supportive care. Overall, distress appeared to be minimized, and the patient was able to maintain aspects of his usual daily routine for a substantial portion of the CKM period.

The patient remained on conservative kidney management without initiation of dialysis and died peacefully at home 35 months later, surrounded by family.

## Discussion

In the present case, the assessment of the patient’s best interests was grounded in a structured and iterative process rather than a single deliberation. Repeated multidisciplinary team meetings were convened to review the totality of the patient’s lived experience: his observable expressions of comfort and distress, his capacity to participate in meaningful daily activities, and the centrality of familiar routines—particularly his attendance at the daytime workshop—to his overall quality of life. The anticipated burdens of each treatment modality were assessed in concrete terms, including the need for repeated restraint or sedation during hemodialysis, the risk of catheter interference with peritoneal dialysis, and the impossibility of sustaining lifelong immunosuppressive adherence following transplantation. Regarding the role of the parents, their perspectives were incorporated as an essential source of knowledge about the patient’s behavioral cues, daily preferences, and aversions—rather than as autonomous surrogates with sole authority over the final decision. The clinical team carefully evaluated whether parental preferences were consistent with the patient’s observable best interests and ensured that the ultimate decision was anchored in the patient’s own experiential welfare. Uncertainty was openly acknowledged throughout this process, and no single consultation was treated as definitive.

The deliberations that led to the selection of CKM were structured around several key ethical and clinical questions. First, the goals of care were explicitly clarified: whether the primary aim should be life prolongation or preservation of quality of life. Second, the anticipated benefits and burdens of each kidney replacement therapy modality were systematically reviewed, with particular attention to this patient’s inability to understand or cooperate with medical procedures. Third, the long-term sustainability of caregiver-dependent management was carefully evaluated, given the patient’s near-total dependence on his mother for all aspects of daily care. Fourth, formal psychiatric assessment confirmed the absence of decision-making capacity, and guidance from the Japan Pediatric Society guidelines and the national end-of-life decision-making framework informed the ethical legitimacy of the surrogate process. Together, these deliberations provided the clinical and ethical basis for the final decision, and the transparency of this process allowed all stakeholders—clinicians, parents, and extended family—to reach a shared understanding.

This case highlights the complex ethical and clinical challenges involved in kidney therapy decision-making for patients with a lifelong severe intellectual disability who lack decision-making capacity. While conservative kidney management (CKM) has been increasingly recognized as a patient-centered alternative to dialysis in selected populations [1–3], existing frameworks largely assume the presence of a patient capable of participating in shared decision-making (SDM). This assumption limits their applicability in cases such as the present one.

A central issue in this case was the patient’s profound lack of decisional capacity. Importantly, lack of decision-making capacity does not imply the complete absence of patient preferences. Although the patient was unable to understand or communicate treatment choices in a legally valid manner, he demonstrated consistent non-verbal expressions of comfort and distress. These behavioral cues, together with knowledge of his daily routines and values, were incorporated into the decision-making process. This distinction is important, as it shifts the ethical focus from a conventional autonomy-based SDM model toward a surrogate-informed and best-interest–oriented process [4, 5].

In practice, however, substituted judgment was difficult to apply because the patient had never been able to form or express complex treatment preferences. Consequently, decision-making relied primarily on a best-interest standard informed by close family members who had longstanding knowledge of the patient’s behaviors, routines, and sources of distress. At the same time, such an approach requires careful attention to the possibility that surrogate preferences may inadvertently overshadow the patient’s interests. For this reason, the decision-making process was iterative, multidisciplinary, and transparent, rather than based on a single conversation or a one-time family preference.

A key ethical tension in this case arose from the discrepancy between technical feasibility and meaningful benefit. All forms of kidney replacement therapy were technically possible; however, each modality was associated with substantial burdens that were unlikely to translate into meaningful improvements in quality of life, reduction of treatment burden, or fulfillment of the patient’s best interests. Hemodialysis would likely have required repeated restraint or sedation, with concerns regarding safety, sustainability, and distress. Peritoneal dialysis posed major challenges related to catheter safety, treatment adherence, and caregiver dependence. Kidney transplantation, while potentially life-prolonging, was not considered sustainable because perioperative management and long-term adherence to immunosuppressive therapy were unlikely to be achievable. These considerations are consistent with clinical and ethical guidance recognizing that dialysis is not obligatory when anticipated burdens outweigh potential benefits [6–8].

This case also illustrates the importance of evaluating quality of life in a manner appropriate to the patient’s cognitive and social context. For this patient, quality of life was closely tied to continuity of familiar routines, including attending his daytime workshop, living at home with his parents, and avoiding distressing medical environments. Interventions that might prolong survival but significantly disrupt these elements were therefore considered to carry a disproportionate burden. This interpretation is concordant with prior reports showing that CKM may reduce treatment burden while preserving quality of life in selected populations [1–3, 9, 10], and with broader literature emphasizing the existential and supportive care needs of patients with advanced kidney disease [11–13].

Another important consideration is the variability in ethical and legal frameworks across healthcare settings. In some regions, individual autonomy is emphasized more strongly, whereas in others, family-centered deliberation plays a larger role. Similarly, SDM in kidney failure has increasingly been reconceptualized as an iterative process that can incorporate uncertainty, surrogate involvement, and evolving goals of care [4, 5]. For an international readership, it is therefore important not to overgeneralize from a single institutional or national context. In the present case, given that the patient’s intellectual age was consistent with that of a young child, the decision-making process drew upon pediatric end-of-life guidelines alongside adult nephrology frameworks. Specifically, the Japan Pediatric Society’s revised 2024 guidelines [14] and the national end-of-life care guidelines [15] were consulted, as no nephrology-specific guidance existed for younger patients with a lifelong severe intellectual disability who lack decision-making capacity in Japan.

This case also highlights the profound emotional burden placed on surrogate decision-makers, and the importance of addressing it as an integral component of care. The patient’s parents experienced significant psychological distress throughout the decision-making process, including anticipatory grief as the clinical trajectory became increasingly clear. Longitudinal psychosocial support was provided by a multidisciplinary team comprising physicians, nurses, medical social workers, and mental health professionals. Regular family meetings were held not only to communicate clinical information but also to create structured space for the parents to express their fears, uncertainties, and grief. This support was initiated early in the decision-making process and continued through the patient’s death at home, reflecting the recognition that emotional care for the family is not ancillary but central to ethically sound CKM. This approach is consistent with palliative care principles and with the growing recognition that surrogate decision-makers require sustained psychological support, particularly when foregoing life-prolonging therapy [16].

From a bioethical standpoint, the present case can be understood through the four-principles framework of respect for autonomy, beneficence, non-maleficence, and justice [17]. Because the patient lacked the capacity to make a treatment decision, autonomy could not be applied in the usual decisional sense; however, respect for the person still required attention to his observable preferences, aversions, and lived experience. Beneficence and non-maleficence required careful weighing of whether technically feasible treatment would provide meaningful benefit or instead impose disproportionate suffering. In addition, the clinical conclusion that the patient lacked decisional capacity was consistent with widely accepted criteria requiring the ability to express a choice, understand relevant information, appreciate one’s situation, and reason about treatment options [18].

Although the timeline presented in Table 1 reflects the course of a single patient, it was not the result of an ad hoc process but rather an intentionally structured approach to decision-making. Key elements included early initiation of discussions regarding kidney replacement therapy, repeated multidisciplinary meetings, involvement of surrogate decision-makers, and iterative reassessment of goals of care. While the specific clinical and social context may not be generalizable, this structured and longitudinal decision-making process may provide a reproducible framework for managing similar cases involving patients with profound lack of decision-making capacity.

**Table 1:** Timeline of clinical events and family discussions leading to the selection of CKM

Timeline (Date)	Clinical Findings	Clinical Interventions	Family Discussions/ Interventions	Outcomes
January 202X, Week 1	S-Cr: 3.8 mg/dL eGFR: 15.9 mL/min/1.73 m²	Initial CKM discussion; RRT options introduced	Parents expressed initial hesitation regarding dialysis	Continued monitoring planned
January 202X, Week 4	S-Cr: 3.9 mg/dL eGFR: 15.4 mL/min/1.73 m²	Follow-up outpatient visit to reassess kidney function	Family expressed interest in learning more about CKM	Educational session on QOL planned
February 202X, Week 3	S-Cr: 4.0 mg/dL eGFR: 15.0 mL/min/1.73 m²	CKM and RRT feasibility compared	Parents leaning toward CKM; MSW introduced for support	Decision-making framework established
March 202X, Week 2	S-Cr: 4.2 mg/dL eGFR: 14.2 mL/min/1.73 m²	Multidisciplinary team reviewed CKM implementation	Siblings consulted remotely; psychological counseling provided	Plan for further discussion at next visit
April 202X, Week 1	S-Cr: 4.3 mg/dL eGFR: 13.8 mL/min/1.73 m²	Detailed QOL assessment completed	Family voiced preference for CKM but requested time to confirm	Next steps aligned with CKM pathway
May 202X, Week 2	S-Cr: 4.5 mg/dL eGFR: 13.2 mL/min/1.73 m²	Final pre-CKM review conducted	Agreement reached among family members; MSW facilitated discussions	CKM confirmed
June 202X, Week 1	S-Cr: 4.7 mg/dL eGFR: 12.6 mL/min/1.73 m²	Home care framework initiated	Parents trained for home-based end-of-life care	CKM implementation initiated
July 202X, Week 2	S-Cr: 4.85 mg/dL eGFR: 12.1 mL/min/1.73 m²	CKM progress evaluation	Family satisfaction assessed; anticipatory grief support continued	CKM ongoing; family support ongoing

CKM, conservative kidney management; eGFR, estimated glomerular filtration rate (mL/min/1.73 m²); MSW, medical social worker; QOL, quality of life; RRT, renal replacement therapy; S-Cr, serum creatinine (mg/dL)

Taken together, this case suggests that CKM may represent an ethically appropriate and clinically meaningful option for younger patients with advanced CKD who lack decisional capacity, particularly when kidney replacement therapies are technically feasible but unlikely to provide proportional benefit. Rather than being viewed as the absence of treatment, CKM should be understood as an active care pathway integrating symptom management, family support, and ongoing reassessment of goals of care.

## Conclusion

This case demonstrates that conservative kidney management can be an ethically and clinically appropriate option for younger patients with advanced CKD who lack decisional capacity. Kidney therapy decisions should extend beyond age and technical feasibility to incorporate treatment burden, quality of life, and the preservation of a coherent lived experience. Integrating surrogate-based decision-making frameworks, evidence-informed CKM practices, and an existentially informed perspective may help guide kidney care for vulnerable populations not adequately addressed by traditional dialysis-centered models.

## Data Availability

The data underlying this article will be shared on reasonable request to the corresponding author.
